# Frequency and distribution of mixed *Plasmodiumfalciparum*-*vivax* infections in French Guiana between 2000 and 2008

**DOI:** 10.1186/s12936-015-0971-1

**Published:** 2015-11-10

**Authors:** Marine Ginouves, Vincent Veron, Lise Musset, Eric Legrand, Aurélia Stefani, Ghislaine Prevot, Magalie Demar, Félix Djossou, Paul Brousse, Mathieu Nacher, Bernard Carme

**Affiliations:** Medicine Department, Ecosystemes Amazoniens et Pathologie Tropicale, EA 3593, Labex CEBA, University of French Guiana, Cayenne, French Guiana; Laboratoire de Parasitologie, Centre Collaborateur OMS pour la surveillance de la résistance aux antipaludiques, CNR du Paludisme, Institut Pasteur de la Guyane, Cayenne, French Guiana; Research Unit of Genetics and Genomics of Insect Vectors, Institut Pasteur, Paris, France; Unité des Maladies Infectieuses et Tropicales, Centre Hospitalier de Cayenne, Cayenne, French Guiana; Pôle des Centres Délocalisés de Prévention et de Soins, Centre Hospitalier de Cayenne, Cayenne, French Guiana; Centre d’Investigation Clinique Epidémiologie Clinique Antilles Guyane CIC EC 1424, Cayenne General Hospital, Cayenne, French Guiana

**Keywords:** Mixed infections, *Plasmodium vivax*, *Plasmodium falciparum*, Treatment, French Guiana

## Abstract

**Background:**

The two main plasmodial species in French Guiana are *Plasmodium vivax* and *Plasmodium falciparum* whose respective prevalence influences the frequency of mixed plasmodial infections. The accuracy of their diagnosis is influenced by the sensitivity of the method used, whereas neither microscopy nor rapid diagnostic tests allow a satisfactory evaluation of mixed plasmodial infections.

**Methods:**

In the present study, the frequency of mixed infections in different part of French Guiana was determined using real time PCR, a sensitive and specific technique.

**Results:**

From 400 cases of malaria initially diagnosed by microscopy, real time PCR showed that 10.75 % of the cases were mixed infections. Their prevalence varied considerably between geographical areas. The presence, in equivalent proportions, of the two plasmodial species in eastern French Guiana was associated with a much higher prevalence of mixed plasmodial infections than in western French Guiana, where the majority of the population was Duffy negative and thus resistant to vivax malaria.

**Conclusion:**

Clinicians must be more vigilant regarding mixed infections in co-endemic *P.* *falciparum/P.* *vivax* areas, in order to deliver optimal care for patients suffering from malaria. This may involve the use of rapid diagnostic tests capable of detecting mixed infections or low density single infections. This is important as French Guiana moves towards malaria elimination.

## Background

In the early 2000s, French Guiana was one of the most malaria-affected territories among the South American regions [1]. Between 2000 and 2009, the average yearly number of cases was 3920, although, the decline was rapid and marked thereafter to reach only 445 cases in 2014 [2]. The dominant plasmodial species are *Plasmodium vivax* and *Plasmodium falciparum.Plasmodium malariae* is much rarer, only representing 1 % of cases [3, 4]. For the past 30 years, malaria transmission nearly no longer affects the coastal area of French Guiana [5], but it persists in the interior regions [6]. In these areas, where only 15 % of the 230,000 inhabitants of French Guiana live, infections were mainly observed among populations living along the Maroni River in the western part of French Guiana bordering Suriname, and the Oyapock in the eastern part bordering Brazil. Nowadays, infections are mainly related to mining activities [7].

The presence of *P. falciparum* was more frequent in western French Guiana, where the Maroon populations live. This population is resistant to *P. vivax* because, as most Africans, they do not express the Duffy antigen. Eastern French Guiana was mostly populated by Amerindians and the incidence of *P. vivax* is similar to that of *P. falciparum* [8]. During the 2000s, there has been an increase in *P. vivax* malaria in eastern French Guiana and a decrease of *P. falciparum* malaria in western French Guiana, notably along the Maroni River [3]. Overall, the proportion of *P. falciparum* in French Guiana has thus decreased from 46 % of all malaria cases in 2005 to 30 % in 2014 [2, 9].

The specific treatment used in French Guiana depends on the infecting species, the severity of disease and the patient’s condition. Chloroquine is administered in uncomplicated vivax malaria, in association with primaquine. For *P. falciparum*, artemether and lumefantrine (Riamet^®^) is employed since 2007. Thus, the identification of the infecting plasmodial species is essential to select the appropriate treatment.

Although errors in species identification are rare, it is common to miss mixed species infections by the microscopic examination of blood smears, particularly when one of the species is predominant in the patient’s blood, which is a frequent situation [10, 11]. In addition, most rapid diagnostic tests, and notably the one used in French Guiana (SD Bioline^®^ Pf/Pan), cannot distinguish between single *P. falciparum* infections and a mixed *P.* *falciparum*/*P. vivax* infections. This may lead to inadequate treatment, since misdiagnosis of a *P.falciparum/P. vivax* mixed infection as a *P. falciparum* infection may lead to failure to administer primaquine and hence will lead to vivax relapses, while misdiagnosis of *P. falciparum/P. vivax* as *P. vivax* infection may lead to the use of chloroquine for resistant, potentially severe, *P. falciparum* infections. Therefore, it is important for clinicians to have local data on the frequency of mixed infections. This problem concerns areas with resistant *P. falciparum* and/or *P. vivax* with frequent relapses, which was the case in French Guiana [12, 13]. It is thus important to use more sensitive and discriminant techniques such as PCR [14–19].

In the present study, the frequency of mixed *P.falciparum/P. vivax* infections in eastern and western French Guiana were thus determined using real-time PCR and compared to microscopic results.

## Methods

### Samples

Between 2000 and 2008, malaria diagnoses in the health centres and at the Hospital were performed using thin and thick blood smears, and were then confirmed by an experienced microscopist at the Department of Parasitology and Mycology at the Hospital. Given the low prevalence of mixed infections using microscopy, PCR diagnosis was implemented in order to estimate the prevalence of mixed infections.

The study included samples from patients with clinical malaria having consulted remote health centres or Cayenne Hospital between 2000 and 2008. Samples were collected for diagnostic purposes on filter paper (Whatman^®^) or in EDTA vacutainers, through the Cayenne Hospital or the National Reference Centre for Malaria, respectively. When filter paper was available and when the physician on site was informed (fast rotation of professionals in these remote centers, with newcomers not aware of all ongoing protocols) the filter paper was sent to the Hospital where PCR was performed. Thus, whether a patient eventually got PCR, it was not linked to the particulars of the patient or the malaria episode leading to the consultation, but to the health care professional rotation. Thus, this was not likely to be a recruitment bias.

**Fig. 1:**
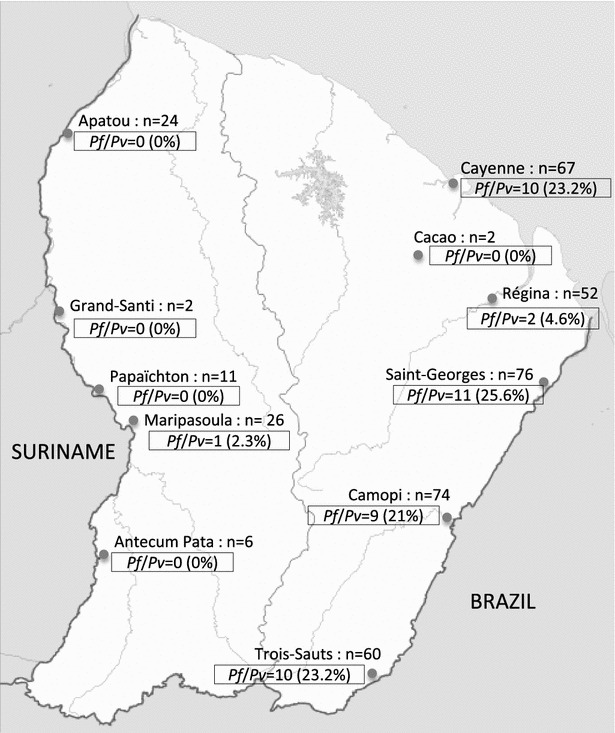
Distribution of mixed infections from different parts of French Guiana

Among the 400 samples collected (representing 10 % of French Guiana cases), 331 came from health centres located in the eastern part of French Guiana: Cacao, Saint-Georges-de-l’Oyapock, Régina, Camopi, Trois-Sauts, and Cayenne Hospital (Fig. 1). In the latter, samples came from patients living in Cayenne or in other towns of central or eastern French Guiana.

The 69 other samples came from health centres located in the western part of French Guiana: Antecume Pata, Maripasoula, Papaïchton, Grand-Santi, and Apatou (Fig. 1). All samples were anonymized before transmission.

### DNA extraction

The DNA from the samples conserved on filter paper or at −20 °C were extracted using the DNeasy^®^ Blood and Tissue Kit or the Qiaamp DNA blood kit (Qiagen, Crawley, UK), as recommended by the manufacturer.

### Real-time PCR

DNA was amplified using real time PCR as described by Veron et al. [20]. The amplified region corresponds to the small 18S RNA sub-unit. The *P. falciparum* primer sense sequence was: Pf1 5′-ATTGCTTTTGAGAGGTTTTGTTACTTT-3′, that of the antisense primer was: Pf-2 5′-GCTGTAGTATTCAAACACAATGAACTCAA-3′ and that of the probe was: Pf-probe FAM-CATAACAGACGGGTAGTCAT-MGB. The size of the amplicon was 95 pb. The sequence of the *P. vivax* sense primer was: Pv-1 5′-CGCTTCTAGCTTAATCCACA TAACTG-3′, that of the antisense primer was: Pv-2 5′-AATTTACTCAAAGTAA CAAGGACTTCCAAG-3′ and that of the probe was: Pv-probe VIC-CGCATTTT GCTATTATGT-MGB. The amplicon size was 142 pb. The amplification and the detection of DNA were performed in duplex, using the PCR Applied Biosystem 7300 analytic system. Real-time PCR was performed in a final volume of 25 µl, in presence of an internal positive control (IPC) (Applied Biosystem, Courtaboeuf, France). Samples were analysed in two tubes, the first one contained *P. falciparum* and *P. vivax* primers and probes and the second one the internal control of the absence of inhibitor, the IPC. Each reaction mix contained 12.5 µl of Master Mix Gene Expression (Applied Biosystem), 300 nM of each primer (*P. falciparum* and *P. vivax*), 150 nM of each probe (*P. falciparum* and *P. vivax*) and 5 µl of matrix DNA. The IPC was used as recommended by the manufacturer. PCR starts with a 10-min phase at 95 °C, followed by 50 cycles of 15 s at 95 °C and 1 min at 60 °C.

### Statistical analysis

Proportions of mixed infections were compared between regions using the χ^2^ test.

### Ethical consideration

The retrospective use of anonymous patient files on the site of patient care is authorized by the French National Commission on Informatics and Liberties (CNIL). All the human blood samples and the data collected retrospectively were anonymized in a standardized case report form and in database.

## Results

The quantities of the different plasmodia species detected for microscopy and real-time PCR are presented in Table 1. The real-time PCR results (Table 1) showed the presence of 149 *P. falciparum* and 140 *P. vivax* infections in the eastern French Guiana and 62 *P. falciparum* and 6 *P. vivax* infections in western French Guiana. Forty-three samples, within the 400 collected samples, corresponded to mixed *P. falciparum*/*P. vivax* infections, which represented a frequency of 10.75 %, versus 2 % in microscopy. Forty-two originated from Eastern French Guiana and only one came from the western part. The latter was an isolate from Maripasoula, a municipality which marks the end of the Maroon territory and the beginning of Amerindian territory. Thus, in eastern French Guiana the proportion of mixed infections was 12.7 % whereas in western French Guiana the prevalence was only 1.4 %. This difference was statistically significant (P < 0.0001). The municipalities of Saint-Georges-de-l’Oyapock (n = 11), Camopi (n = 9), Trois-Sauts (n = 10), and Cayenne (n = 10) were the sites with the highest number of mixed infections observed (Fig. 1).

**Table 1:** Microscopy and real-time PCR results

	*P. falciparum*		*P. vivax*		Pf/Pv		Total
	Microscopy	Q-PCR	Microscopy	Q-PCR	Microscopy	Q-PCR	
East	169 (51.1 %)	149 (45.0 %)	155 (46.8 %)	140 (42.3 %)	7 (2.1 %)	42 (12.7 %)	331
West	62 (89.9 %)	62 (89.9 %)	6 (8.7 %)	6 (8.7 %)	1 (1.4 %)	1 (1.4 %)	69
Total	231 (57.75 %)	211 (52.75 %)	161 (40.25 %)	146 (36.5 %)	8 (2.0 %)	43 (10.75 %)	

*Pf/Pv* the number of cases of mixed infections *P. falciparum/P. vivax* detected by real-time PCR for each remote health centres. The percentages of each species of plasmodium and mixed infections Pf/Pv were shown in parentheses

Mixed infections misdiagnosed by microscopy were only observed in eastern French Guiana. In 15 cases the association was diagnosed as *P. vivax* and in 20 cases, as *P. falciparum* (Table 1).

## Discussion

Overall, mixed plasmodial infections were frequent in French Guiana with 10.75 % of malaria cases having mixed *P.vivax/P. falciparum* malaria. This overall figure however, masks a very heterogeneous situation between eastern, where most mixed infections came from, and western French Guiana, where there was only 1.4 % mixed infection. The incidence of *P.vivax* malaria is very low among Maroon populations, who are Duffy negative and the main ethnic group living on the Maroni River. This ethnic particularity could explain the low prevalence of mixed infections in western French Guiana [21, 22].

Other authors have observed that over a quarter of *P. falciparum* infections were in fact mixed infections [23]. Studies conducted in different endemic areas also had different designs often involving cross-sectional studies of exposed populations, and not microscopically confirmed malaria patients as in this study. Molecular studies from Brazil showed that *P. falciparum* mixed species were detected in 30 % [24], 23.4 % [25] and 10 % [26] in Rondônia for the first two, and Apiacas, respectively. The proportion of mixed infections was lower in Brazil than in some studies from Thailand (24.2–51.6 %) [27] and Papua New Guinea (65.3 %) [17], and similar in Laos (23.1 %) [28].

Mixed infections have been associated with less severe malaria by some [29, 30] and with severe malaria [31] or higher fever [32] by other authors. Apart from the immunologic and pathophysiologic consequences of mixed infections, their misdiagnosis could lead to treatment that is not effective against the hidden species. Thus, missing a hidden *P. falciparum* infection leads to treatment with chloroquine with potential risks for the patient owing to the 25 % of chloroquine-resistant parasites circulating in the region [33]. Conversely, when *P. vivax* is hidden, although artemisinin-based combination therapy (ACT) will kill *P. vivax*, treatment of latent hypnozoites with primaquine will be omitted thus leading to the risk of *P. vivax* relapses, notably as *P. falciparum* malaria re-activates latent hypnozoites [13, 34, 35]. As the French and Brazilian Ministers of health announced in July 2015, malaria elimination was a common goal, the capacity to diagnose low density infections is capital [36]. However, when comparing the cases of mixed infections in 2006 and 2007, there was a significant decrease, respectively 18.7 and 9.8 % (p = 0.03), presumably following the overall incidence decrease.

PCR is much more sensitive than microscopy and rapid diagnostic tests, notably to detect mixed infections [14–19]. However, the high costs of this technique are still an obstacle for its use in remote health centres of French Guiana. Nevertheless, depending on the microscopist experience, TDR could be a more efficient technique than microscopy [37] and could be applied to remote areas, provided it is sufficiently sensitive to detect the two *Plasmodium* species at low parasite densities.

## Conclusion

Microscopy often fails to reveal mixed infections or low density single infections. In French Guiana, there is a singular situation with inhabitants of different ethnic origins and therefore malaria susceptibilities, and with differences in local epidemiology, which should be known by clinicians who in routine care do not have access to real-time PCR and thus have a non-negligible risk of overlooking mixed infections. The detection or the anticipation of mixed *P.vivax/P. falciparum* infections is of clinical importance because interactions between the different species simultaneously infecting the same patient could result in significant changes in the course of the infection and disease, and thus affect therapeutic strategies. The development of rapid diagnostic tests for the detection of mixed infections or the systematic and simultaneous use of two rapid diagnostic tests allowing: (1) the detection of low densities of *P. vivax*, and, (2) the detection of low densities of *P. falciparum*, to improve mixed infections diagnosis and treatment, and further decrease malaria as French Guiana moves towards malaria elimination.
